# Analysis of risk factors for postoperative recurrence of stage IA lung adenocarcinoma

**DOI:** 10.3389/fonc.2025.1557081

**Published:** 2025-08-11

**Authors:** Wendong Xu, Runze Li, Dilihumaer Tuerxun, Yuanguo Wang, Jian Li, Jingyu Li, Peng Zhang

**Affiliations:** ^1^ Deparment of Cardiothoracic Surgery, Tianjin Medical University General Hospital Airport Hospital, Tianjin, China; ^2^ Deparment of Cardiothoracic Surgery, Tianjin Medical University General Hospital, Tianjin, China

**Keywords:** lung adenocarcinoma, recurrence, competing risk analysis, vascular invasion, postoperative recurrence

## Abstract

**Objective:**

To explore the influencing recurrence in patients with stage IA lung adenocarcinoma following surgical resection.

**Method:**

A retrospective analysis was conducted on the clinical and pathological data of patients with stage IA lung adenocarcinoma who underwent surgical resection in the Department of Thoracic Surgery at Tianjin Medical University General Hospital from January 1, 2018, to December 31, 2021. The Fine-Gray model was utilized for multivariate analysis to identify factors influencing the cumulative incidence of lung cancer recurrence. The hazard ratio (HR) and its 95% confidence interval (CI) were calculated.

**Result:**

Among 475 patients, there were 30 cases of postoperative recurrence and metastasis. The results of the univariate analysis using the Gray test indicated that vascular invasion, STAS, solid and micropapillary patterns, pTNM classification, lymph node resection method, surgical method, organizational differentiation, CTR had a significant impact (P<0.05). The multivariate analysis revealed that pTNM classification, lymph node resection method, vascular invasion and the presence of solid or micropapillary patterns were significantly correlated.

**Conclusion:**

Tumor staging, the presence of solid or micropapillary components in pathology, vascular invasion, and the method of lymph node resection significantly influence postoperative disease-free survival (DFS) in patients with stage IA lung adenocarcinoma undergoing surgical resection.

## Background

Lung cancer has the highest incidence and mortality rates among malignant tumors, and these rates are increasing rapidly each year, making it a significant public health concern (1). According to GLOBOCAN’s 2022 data, there are 20 million new cancer cases and 9.7 million cancer-related deaths globally each year, with lung cancer contributing 2.5 million new cases and 1.8 million deaths. This represents 12.4% of all cancer cases and 18.7% of cancer deaths for that year (2). The decline in the number of smokers, coupled with advancements in early detection and treatment strategies, has contributed to a year-on-year reduction in cancer mortality rates in developed nations. The 2022 US Cancer Statistics Report indicates that from 1991 to 2019, the overall cancer mortality rate in the United States experienced a 32% decrease, primarily attributed to a reduction in lung cancer mortality (3). Conversely, in China, the incidence of lung cancer is increasing, driven by factors such as the rapid rise in smoking rates, air pollution, and an aging population. Data from the China Cancer Registration Annual Report, covering the years 2000 to 2018, reveals that lung cancer incidence is the highest among 28 provinces, autonomous regions, and municipalities, with a notable trend towards younger demographics (4). The incidence rate of lung cancer among young patients in China stands at 12.1%, surpassing that of their counterparts in the United States.

The advancement of multi-slice spiral computed tomography technology, coupled with the widespread adoption of low-dose spiral CT screening, has led to an increased detection of early-stage lung adenocarcinoma manifesting as ground glass nodules (GGNs) (5). Research indicates that between 2010 and 2019, there has been a rise in the proportion of lung cancer diagnosed locally among both males and females in the United States, with an average annual percentage change of 4.9% for males and 4.5% for females. This data suggests a growing trend in the early diagnosis of lung cancer (6). From a global standpoint, the evolution of lung cancer screening technologies, particularly the extensive utilization of low-dose computed tomography (LDCT), has significantly enhanced the early detection rates of lung cancer. The National Lung Screening Trial conducted in the United States has robustly validated the efficacy of LDCT in screening populations at high risk for lung cancer. Subsequent research has consistently demonstrated that in regions where LDCT screening is implemented, there is a marked increase in the incidence of early lung cancer diagnoses (7). In China, the increasing adoption of low-dose chest CT for screening high-risk individuals has led to a rise in the number of early lung cancer cases. Furthermore, the “Chinese Expert Consensus on Early Lung Cancer Diagnosis,” published in 2023, highlighted that the widespread use of high-resolution CT and related technologies has significantly improved the detection rates of small lung nodules, particularly ground-glass nodules, a substantial proportion of which are identified as early-stage lung cancer. This trend further substantiates the potential for identifying an increasing number of early-stage lung cancer patients (8). In the findings reported by Maeda et al., the 5-year survival rates for stage IA and IB lung cancer were documented at 89.9% and 72.3%, respectively (9). Furthermore, Goldstraw noted variations in the survival prognosis among stage I lung cancer patients, with 5-year survival rates of 90%, 85%, 80%, and 73% for stages IA1, IA2, IA3, and IB, respectively (10).

Recurrence is a significant determinant of postoperative survival among patients. The postoperative recurrence rate for stage I non-small cell lung cancer (NSCLC) can reach as high as 36% (11). Currently, the findings regarding this issue are inconsistent across both domestic and international studies. For instance, Wang et al. conducted a study involving 1,387 patients with stage I lung cancer who underwent radical surgical intervention, reporting a recurrence rate of 21.7% among 301 patients (12). Conversely, in the research conducted by Zhang Yang et al., which focused on 1,108 patients with stage I invasive lung adenocarcinoma (excluding micro-invasive and adherent types) and squamous cell carcinoma who underwent complete surgical resection, a total of 184 patients experienced recurrence, resulting in a recurrence rate of 15.6% (13). Currently, the majority of research focuses on the postoperative recurrence of stage IA non-small cell lung cancer, with a significant portion of this literature lacking stratification (9). Furthermore, there is a scarcity of studies examining the factors associated with postoperative recurrence specifically in stage IA lung adenocarcinoma. The 8th edition of the American Joint Committee on Cancer (AJCC) has delineated stage IA into three subcategories: IA1 (tumor size ≤1 cm), IA2 (tumor size 1–2 cm), and IA3 (tumor size 2–3 cm). However, most studies have not performed stratified analyses for these sub-stages, which obscures the prognostic distinctions related to varying tumor diameters. Additionally, there is an absence of standardized assessments regarding pathological characteristics, such as the proportion of microinvasive lesions, the presence of STAS, and vascular invasion. For instance, patients exhibiting STAS positivity may experience a recurrence risk that is 2–3 times greater; however, the inclusion rate of such patients in existing studies is below 30%. We conducted a retrospective analysis of data from 459 patients diagnosed with stage IA lung adenocarcinoma who received surgical intervention at the Department of Thoracic Surgery, Tianjin Medical University General Hospital, between January 1, 2018, and December 31, 2021. Utilizing statistical methodologies, we aimed to identify factors influencing the recurrence of lung adenocarcinoma in this patient cohort post-surgery, thereby offering insights that may enhance the survival outcomes for individuals with stage IA lung adenocarcinoma. This research elucidated the significance of high-risk factors for recurrence in categorizing the risk of recurrence among patients with stage IA lung adenocarcinoma following surgical intervention. The findings offer a foundation for postoperative management strategies, which may include enhanced monitoring, increased frequency of follow-up appointments, or the consideration of adjuvant therapy.

## Patients and methods

### Patients and study design

This research involved a cohort of 475 patients who received surgical intervention within the Thoracic Surgery Department at Tianjin Medical University General Hospital from January 1, 2018, to December 31, 2021. The inclusion criteria for this study were as follows: (1) Patients who underwent surgical procedures such as lobectomy, segmental resection, or wedge resection; (2) All patients had comprehensive preoperative evaluations, which included chest and abdominal computed tomography (CT) or abdominal ultrasound, head CT or magnetic resonance imaging (MRI), and bone scans or positron emission tomography-computed tomography (PET-CT), with the exception of those exhibiting distant metastasis; (3) Postoperative pathological assessment confirmed a diagnosis of stage IA lung adenocarcinoma; (4) Patients were required to participate in regular follow-up examinations at either our institution or a local healthcare facility following surgery. The exclusion criteria were as follows: (1) Postoperative pathology indicating minimally invasive adenocarcinoma; (2) Postoperative pathological staging exceeding stage IA; (3) A history of comorbidities involving other malignant tumors; (4) Presence of dual primary or multiple primary cancers; (5) Severe postoperative complications or mortality resulting from complications; (6) Incomplete clinical data; (7) Patients with a follow-up duration of less than 5 months.

The clinical characteristics of the patients participating in this study include gender, age, pTNM stage, method of lymph node resection, number of lymph nodes resected, smoking history, presence of vascular invasion, STAS (Spread Through Air Spaces), histological pattern (solid or micropapillary), surgical technique, Consolidation Tumor Ratio (CTR), organizational differentiation, and body mass index (BMI). This research develops a competitive risk model that integrates both the recurrence status of patients and the duration from surgery to recurrence, facilitating both univariate and multivariate analyses.

### Data collection and definitions

This study involves a retrospective analysis of clinical medical records obtained from electronic medical record systems, encompassing various patient demographics such as age, gender, smoking history, medical history, preoperative imaging, laboratory examination data, surgical records, and postoperative pathological information. The imaging data will be assessed and documented by a minimum of two senior thoracic radiologists, who will also record the proportion of solid nodules. Additionally, the pathological review will be conducted by at least two senior pathologists. Pathological staging, histological classification, and histopathological grading will adhere to the UICC/AJCC 8th edition TNM staging guidelines for lung cancer, as well as the World Health Organization (WHO) guidelines. The study was approved by the Tianjin Medical University General Hospital Ethical Committee (Ethical NO.IRB2024-WZ-01).

Recurrence is defined as any local recurrence or distant metastasis identified through pathology or imaging. In instances where pathological evidence of recurrence is unavailable, cases will be evaluated by multidisciplinary teams comprising experts in thoracic surgery, oncology, imaging, and pathology. The period of recurrence-free survival is defined as the interval from postoperative day one until the diagnosis of recurrence. The follow-up period is set to conclude on May 31, 2024, and all patients have completed their follow-up assessments.

### STAS evaluation criteria

According to the consensus publication on lung adenocarcinoma pathology released in 2021 by the International Association for the Study of Lung Cancer (IASLC), the American Thoracic Society (ATS), and the European Respiratory Society (ERS) (14), the term “STAS” is defined as the presence of tumor cells, either individually or in clusters, within the alveolar cavity, situated at a distance from the periphery of the primary tumor, and lacking any direct association with the main tumor mass. The interpretation process involves utilizing low magnification (×40) to identify the tumor boundary, followed by high magnification (×200) to verify the presence of tumor cells within the alveolar cavity. It is essential to exclude any invasive growth of the tumor or residual margins, and to only consider disseminated lesions that are located at least 0.5 mm from the tumor boundary.

Classification criteria for solid and micropapillary subtypes are delineated in the WHO 2021 classification of lung tumors (15). The solid type is characterized by tumor cells that proliferate in a solid nest-like formation, devoid of glandular or papillary structures, and lacking intracellular mucus. In contrast, the micropapillary type is defined by the presence of tumor cells that aggregate into small clusters of papillary structures, which do not possess a fibrous vascular axis. These clusters are typically observed floating within the alveolar cavity or interstitial space and are often arranged in a back-to-back configuration.

### Follow-up and recurrence treatment

#### The initial day following

surgery is designated as the commencement of the follow-up period, which primarily employs outpatient consultations and telephone communications as its main methods. Patient data is monitored through records from outpatient follow-ups and examination results. For a duration of two years post-surgery, follow-up assessments are performed every three months, encompassing various examination components such as complete blood counts, biochemical blood analyses, tumor marker evaluations, enhanced chest computed tomography (CT), neck ultrasounds, abdominal ultrasounds, brain magnetic resonance imaging (MRI), bone scans, and/or positron emission tomography/computed tomography (PET/CT), among others. Subsequently, routine follow-ups are scheduled biannually for a period of two to five years following the surgical procedure. Criteria for the termination of follow-up include: 1) patient mortality; 2) the final follow-up date (May 31, 2024); and 3) voluntary withdrawal by the patient. Treatment options for recurrence encompass radiotherapy, chemotherapy, immunotherapy, and reoperation.

### Statistical analysis

Utilize the Fine and Gray model to estimate the sub-distribution hazard ratio (SHR) for each factor associated with recurrence, considering non-cancer mortality as the competing event. The assumptions of the model include the independence of the target event from the competing event and the hypothesis of proportional sub-distribution hazards. Independence should be verified through Gray’s test, while the proportional hazards hypothesis can be validated using the Grambsch-Thorneau test. The findings will be presented as SHR along with a 95% confidence interval, and a visual analysis will be conducted in conjunction with the cumulative incidence curve. In this study, deaths attributed to non-cancer causes, such as cardiovascular disease, pneumonia, and trauma, are classified as competing events and are excluded from the study endpoints. A univariate analysis will be performed using the cumulative incidence function (CIF) to illustrate the probability of each event occurring, and Gray’s test will be employed to assess the differences in CIF across groups. The Fine and Gray model will also be applied for multivariate analysis to identify factors influencing the cumulative incidence of lung cancer recurrence. The hazard ratio (HR) and its corresponding 95% confidence interval (CI) will be calculated. Additionally, a comparison will be made between the results obtained from the Cox regression model and the Fine and Gray model, emphasizing the relevance of the competing risk model in this context.

## Results

**Figure 1:**
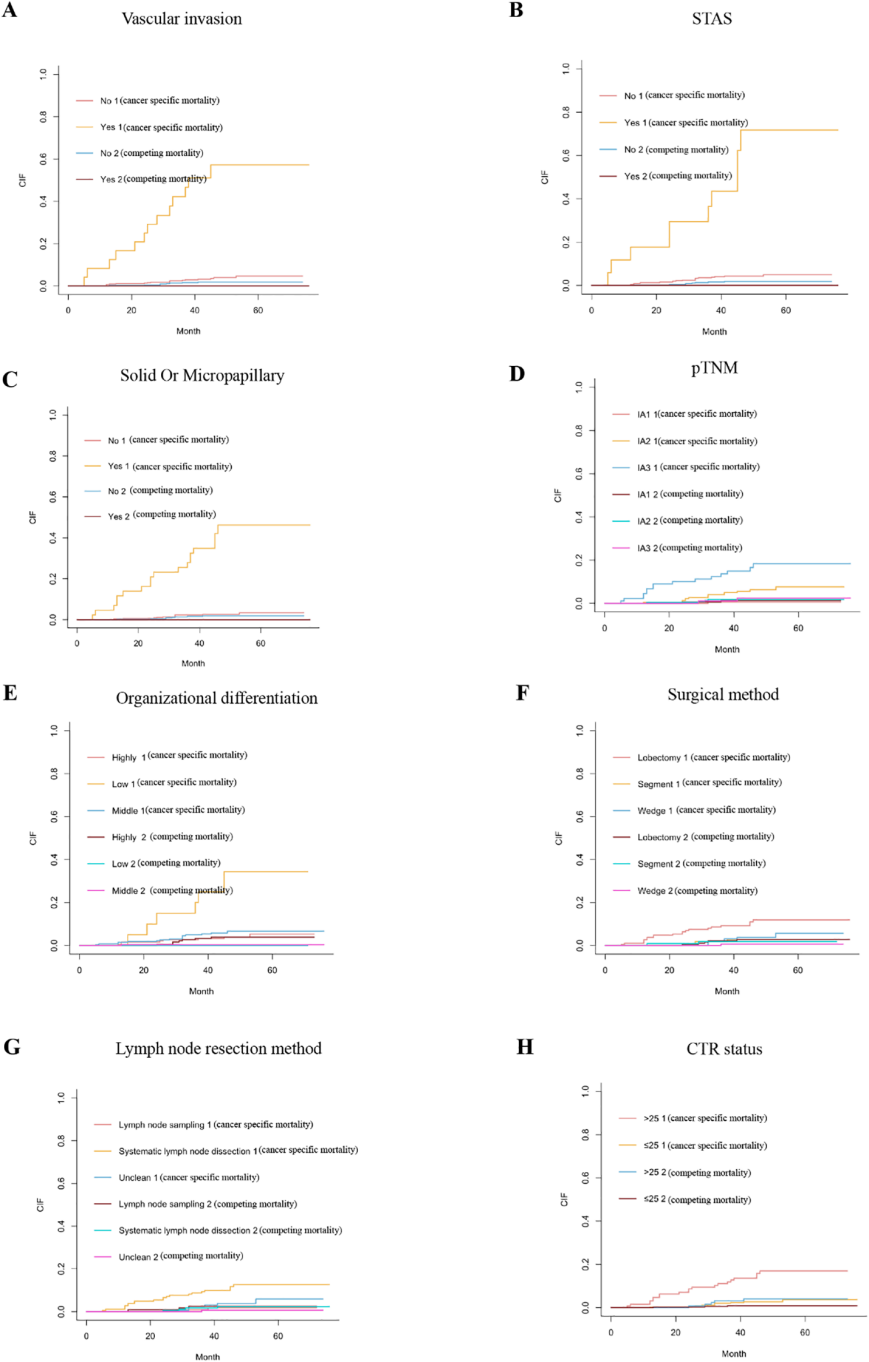
Presents the cumulative disease-free survival (DFS) curves specific to cancer (1) and those accounting for competing risks (2) for patients categorized according to various clinical characteristics: **(A)** vascular invasion; **(B)** spread through air spaces (STAS); **(C)** histological subtype (solid or micropapillary); **(D)** pathological tumor-node-metastasis (pTNM) classification; **(E)** organizational differentiation; **(F)** surgical approach; **(G)** lymph node resection technique; and **(H)** status of the complete tumor resection (CTR).

This research involved a cohort of 475 patients who underwent surgical resection, all of whom satisfied the established inclusion criteria. Among a cohort of 475 patients, 30 individuals exhibited recurrence, resulting in a recurrence rate of 6.3%. Within this cohort, males constituted 35.2% and females represented 60.9%. The median duration of follow-up was 46 months, with a range spanning from 5 to 76 months. Out of the total 475 patients, 8 individuals (1.68%) succumbed to causes unrelated to cancer, with COVID-19 identified as the cause of these non-cancer-related fatalities. Additionally, 8 patients were lost to follow-up due to various unspecified factors. The univariate analysis employed the Gray test and cumulative incidence function (CIF). In the context of competitive risks, the findings from the univariate analysis utilizing Gray’s test indicated that factors such as vascular invasion, STAS, solid or micropapillary subtype, pTNM classification, lymph node dissection method, surgical approach, histological differentiation, and CTR exhibited statistically significant associations with the recurrence of stage IA lung adenocarcinoma (P<0.05). Nearly all variables demonstrated an increase in CIF at the 1, 3, and 5-year marks. The corresponding CIF curve is illustrated in **Figure 1**, with comprehensive data available in **Table 1**. In the presence of competing events, the statistically significant variables identified in the Fine Gray model during the univariate analysis were subsequently incorporated into the multivariate analysis.

**Table 1:** Univariable analysis by using competing risk model.

Variables	Gray’s test	P-value	Cumulative incidence function		
			20-mo	40-mo	60-mo
Gender					
F	1.508	0.219	0.017	0.041	0.066
M			0.022	0.076	0.086
Vascular invasion					
No	**95.873**		0.011	0.029	0.046
Yes			0.166	0.511	0.572
STAS					
No	**84.142**	**<0.001**	0.013	0.04	0.049
Yes			0.17	0.435	0.718
Number of lymph node resections					
>5	0.136	0.712	0.021	0.054	0.075
≤5			0.015	0.053	0.071
Solid Or Micropapillary					
No	**97.986**	**<0.001**	0.006	0.023	0.033
Yes			0.140	0.349	0.462
pTNM					
IA1	**25.130**	**<0.001**	0.000	0.006	0.006
IA2			0.004	0.050	0.076
IA3			0.089	0.149	0.183
Lymph node resection method					
No lymph node dissection	**14.334**	**<0.001**	0.000	0.031	0.058
Lymph node sampling			0.000	0.017	0.017
Systematic lymph node dissection			0.050	0.099	0.126
**Smoking history**	0.073	0.964			
≤400			0.044	0.066	0.066
>400			0.023	0.060	0.060
No			0.014	0.050	0.070
Surgical method					
Lobectomy	**10.643**	**0.004**	0.048	0.092	0.119
Segment			0.000	0.028	0.028
Wedge			0.000	0.030	0.056
Organizational differentiation					
Highly	**20.004**	**<0.001**	0.016	0.032	0.053
Low			0.050	0.250	0.343
Middle			0.018	0.054	0.066
BMI					
<18.5	2.510	0.285	0.000	0.000	0.000
<25			0.025	0.071	0.083
≥25			0.011	0.033	0.061
Age					
>65	1.536	0.215	0.023	0.072	0.088

≤65			0.016	0.043	0.065
CTR					
≤25	**48.791**	**<0.0001**	0.002	0.023	0.037
>25			0.063	0.136	0.169

F denotes Female, M denotes Male, STAS refers to Spread Through Air Spaces, and CTR stands for Consolidation Tumor Ratio.

The highlighted numbers in the Tables indicate statistically significant differences identified through statistical analysis.

Multivariate analysis indicated that the pTNM classification, the method of lymph node resection, the presence of vascular invasion, and the histological subtype (solid or micropapillary) are significantly correlated with the recurrence of stage IA lung adenocarcinoma. Comprehensive data supporting these findings are presented in **Table 2**.

**Table 2:** Multivariate analysis by using competing risk model.

Variables	HR	95% CI	*P*-value
pTNM			
IA2 VS IA1	**7.457**	**0.980-56.738**	**0.043**
IA3 VS IA1	**12.850**	**1.707-96.710**	**0.012**
Lymph node resection method			
Systemsic Lymph node vs No lymph node dissection	0.984	0.123-7.838	0.990
Lymph node sampling vs No lymph node dissection	**0.047**	**0.006-0.386**	**0.004**
Surgical.method			
Segment vs Lobectomy	11.036	0.633-192.373	0.100
Wedge vs Lobectomy	2.590	0.458-14.655	0.280
Organizational			
Low vs Highly	1.865	0.568-6.129	0.300
Middle vs Highly	1.5647	0.519-4.717	0.430
**CTR**(>25vs ≤25)	2.225	0.749-6.607	0.150
**Vascular invasion** (Yes vs No)	**3.907**	**1.261-12.108**	**0.018**
**STAS** (Yes vs No)	1.921	0.741-4.976	0.180
**Solid Or Micropapillary** (Yes vs No)	**4.577**	**1.475-14.201**	**0.009**

STAS refers to Spread Through Air Spaces, and CTR stands for Consolidation Tumor Ratio.

The highlighted numbers in the Tables indicate statistically significant differences identified through statistical analysis.

## Discussion

Research has indicated that the overall survival rate (OS) following radical surgery is 98.6 ± 6%, with a 3-year OS of 94.1 ± 1.8%. Additionally, the postoperative disease-free survival (DFS) rates at 1 year and 3 years are reported to be 97.5 ± 1.8% and 89.7 ± 2.2%, respectively (16). Minimizing the rate of postoperative recurrence continues to pose a significant challenge for thoracic surgeons. In response to this issue, the present study conducts a retrospective analysis of data from 475 patients diagnosed with stage IA lung adenocarcinoma who received surgical intervention at the Department of Thoracic Surgery at Tianjin Medical University General Hospital between January 2018 and December 2021. Utilizing statistical methodologies, this analysis aims to identify the factors influencing postoperative recurrence in patients with stage IA lung adenocarcinoma, thereby providing a foundation for the development of treatment strategies for this patient population. In our study, the recurrence rate among patients was found to be 6.3%. Several factors contributing to this rate were examined. Notably, the COVID-19 pandemic resulted in a substantial rise in the frequency of chest CT examinations, which in turn led to a marked increase in the identification of early-stage lung cancer cases within our cohort. This surge also corresponded with an elevated incidence of patients diagnosed with invasive stage Ia lung adenocarcinoma.

Utilizing univariate analysis through the Fine & Gray model, our study identified several factors that significantly influence postoperative disease-free survival (DFS) in patients diagnosed with stage IA lung adenocarcinoma. These factors include vascular invasion, STAS, solid or micropapillary histological patterns, pTNM classification, lymph node reaction method, surgical approach, organizational differentiation, and CTR. Subsequently, a multivariate analysis employing the Fine & Gray model was performed on the aforementioned significant factors. The findings indicated that pTNM classification, lymph node reservation method, vascular invasion, and solid or micropapillary histological patterns exert a substantial impact on the postoperative recurrence of stage IA lung cancer.

Previous research has indicated that in patients diagnosed with stage I lung adenocarcinoma, the presence of a pathological subtype predominantly characterized by solid components significantly influences the likelihood of postoperative recurrence, which includes cases following lobectomy and sublobectomy procedures. Specifically, patients exhibiting solid components experience earlier recurrences, which are more frequently associated with extrathoracic and multiple metastatic patterns compared to those with non-solid components (17). Furthermore, in early-stage lung adenocarcinoma patients who undergo traditional lobectomy accompanied by mediastinal lymph node dissection, the pathological subtype primarily composed of solid components continues to serve as an independent predictor of postoperative recurrence (18). Building upon these findings, subsequent analyses by various scholars have revealed that lung adenocarcinoma patients whose tumors are predominantly composed of solid components face a poorer prognosis upon recurrence, with an average survival duration post-recurrence being significantly shorter than that of patients whose tumors are primarily non-solid components (HR:2.40; 95%CI=1.13-5.08,P=0.022) (19). The solid type and micropapillary type represent the pathological subtypes of lung adenocarcinoma associated with the most unfavorable prognosis, suggesting that these subtypes exhibit significant invasiveness and poor outcomes (20). In a retrospective analysis conducted by Yue Zhao et al. involving 1,244 patients diagnosed with lung adenocarcinoma, it was observed that patients with solid components constituting more than 5% of the tumor, but not predominantly solid components, experienced shorter disease-free survival (DFS) and overall survival (OS) (p<0.001; p<0.001) and had a higher incidence of lymph node metastasis (p<0.001) compared to those without solid components (21). Our research similarly indicates that the presence of solid or micropapillary components serves as an independent prognostic factor for postoperative recurrence in patients with stage IA lung adenocarcinoma.

Numerous studies have demonstrated that tumor diameter and the CTR play significant roles in influencing the recurrence rates and survival outcomes of lung cancer patients following surgical intervention (22, 23). Research conducted by Hiroaki Nomori et al. indicated that patients with T1N0M0 stage non-small cell lung cancer (NSCLC) who underwent anatomical lung segmentectomy and had a tumor diameter of less than 2 cm exhibited improved 5-year disease-free survival (DFS) and overall survival (OS) rates compared to those with tumors exceeding 2 cm in diameter (24). Similarly, John M. Varlotto et al. identified a tumor diameter greater than 2 cm as a considerable risk factor for postoperative recurrence following lobectomy (25).

The level of tumor differentiation plays a crucial role in determining the prognosis for patients with non-small cell lung cancer (NSCLC). Specifically, a lower degree of differentiation is associated with a reduced postoperative survival duration for these patients (26). In cases of early-stage NSCLC, the degree of tumor differentiation continues to exert a significant influence on patient outcomes. Retrospective studies have demonstrated that, for stage NSCLC, the degree of tumor differentiation markedly impacts the postoperative recurrence rates, irrespective of whether the tumor diameter is ≤3 cm or <2 cm (27, 28). In a retrospective analysis involving 532 patients with stage IA NSCLC who underwent radical lobectomy and mediastinal lymph node dissection, researchers found that the degree of tumor differentiation significantly influenced both the postoperative recurrence rate (HR=1.925, p=0.006) and overall survival (OS) (HR=1.632, p=0.028) (29).

In patients who have undergone surgery for stage IA lung adenocarcinoma, tumor differentiation does not serve as an independent prognostic factor for postoperative disease-free survival (DFS). This conclusion is derived from a retrospective study with a limited sample size, indicating that further validation through larger-scale randomized controlled trials is necessary.

In stage IA lung adenocarcinoma, the presence of spread through air spaces (STAS) is recognized as a prognostic risk factor, with approximately 26.7% to 32.3% of patients exhibiting this characteristic. The five-year disease-free survival (DFS) rate for patients who undergo radical surgical resection and present with STAS ranges from 70% to 80% (30, 31). Similar to vascular invasion (VI), numerous researchers have observed that the prognosis for patients with stage IA and STAS is comparable to that of patients with stage IB overall (32), prompting further stratified analyses. For instance, Dai et al. identified a statistical threshold in prognosis between stage IA patients with STAS and those with stage IB; however, when T1a and T1b were excluded from the analysis, the survival curves for T1c patients with STAS and those with stage IB nearly overlapped (33). Han et al. proposed a classification system for STAS, designating grade I for cases where the dissemination is closer to the tumor edge and grade II for those further away, based on the distance between the disseminated airways and the tumor margin (34). Their findings indicated that only stage IA patients with grade II STAS exhibited a prognosis equivalent to that of stage IB patients.

Our analysis revealed no significant difference in recurrence rates following sublobar resection among patients with stage IA lung adenocarcinoma accompanied by STAS. This may be attributed to the characteristics of stage IA lung adenocarcinoma (T1a/b/cN0M0), which typically presents with a tumor diameter of ≤3cm, predominantly consisting of either adherent or minimally invasive adenocarcinoma, both of which exhibit slow growth and a low propensity for distant metastasis. Even in the presence of STAS, its dissemination may remain confined to the sublobar resection area, thereby not resulting in clinically detectable metastases. Research indicates that the five-year recurrence rate for patients with stage IA and STAS is approximately 10% to 15%, a figure that does not show statistical significance when compared to patients without STAS (35). This observation may stem from the low tumor burden, suggesting that the pro-metastatic influence of STAS has not been fully substantiated.

Sublobar resection necessitates a margin of at least 2 cm or a margin equivalent to the tumor diameter. If STAS is restricted to within 1–2 cm of the tumor, sublobar resection can effectively excise the air cavity containing STAS, thereby mitigating the risk of local recurrence. A study focusing on stage IA tumors measuring ≤2cm demonstrated that when the resection margin is ≥ 2 cm, the local recurrence rate for patients with STAS is 3.2%, which is not significantly different from the 2.8% recurrence rate observed in patients without STAS, indicating that an adequate resection margin can offset the impact of STAS (36). These findings underscore the necessity of a comprehensive evaluation of the clinical significance of STAS, taking into account tumor size, subtype, degree of differentiation, and other relevant factors. Consequently, treatment decisions should not be based solely on a singular pathological feature but should instead be individualized through multifactorial risk stratification.

This study conducted an analysis to elucidate the roles of vascular invasion (VI), pathological TNM (pTNM) staging, the absence of lymph node dissection, and the presence of solid micropapillary components in the risk stratification of recurrence among patients diagnosed with stage IA lung adenocarcinoma post-surgery. The findings provide a foundation for postoperative management strategies. This approach aligns with the rationale of established early risk assessment guidelines for lung cancer, such as those proposed by the National Comprehensive Cancer Network (NCCN), which advocate for the modification of follow-up intensity based on postoperative pathological high-risk factors (including VI and solid subtype) rather than relying solely on preoperative evaluations. Our research indicates that patients exhibiting positive VI, solid micropapillary components, and advanced pTNM staging who have not undergone lymph node dissection demonstrate a significantly elevated recurrence rate compared to those with negative findings. This underscores the necessity for more vigilant monitoring strategies for this particular cohort. In accordance with the consensus from the International Association for the Study of Lung Cancer (IASLC), it is recommended that patients with positive VI undergo chest computed tomography (CT) scans biannually for the initial three years following surgery, alongside serum tumor marker assessments every three months. Furthermore, for patients with positive VI who also present with solid subtypes or tumor diameters exceeding 2 cm, dynamic monitoring of circulating tumor DNA (ctDNA) may be integrated into the follow-up protocol to facilitate the early detection of micrometastasis. It is important to note that the findings of this study do not endorse the routine application of adjuvant chemotherapy in patients with positive VI; however, a combined analysis with molecular markers may inform future personalized treatment approaches (for instance, the responsiveness of EGFR mutant VI positive tumors to tyrosine kinase inhibitors, which warrants further investigation). This represents a prospective avenue for our ongoing research.

This study acknowledges several limitations. As a single-center retrospective analysis, the sample size may not be representative of the broader population. Furthermore, the study is restricted to patients who have undergone thoracoscopic lobectomy, segmental resection, and wedge resection in recent years, which may result in an underestimation of the recurrence rate for stage IA lung adenocarcinoma patients. Nonetheless, certain variables have been effectively controlled within the study. For instance, the surgical procedures were not all performed by physicians of uniform expertise. Moving forward, we aim to conduct large-scale multicenter prospective studies to enhance the understanding of diagnosis and treatment in this area.

## Data Availability

The raw data supporting the conclusions of this article will be made available by the authors, without undue reservation.
